# Effects of low molecular sugars on the retrogradation of tapioca starch gels during storage

**DOI:** 10.1371/journal.pone.0190180

**Published:** 2017-12-28

**Authors:** Xiaoyu Zhang, Rongfang Li, Huaibin Kang, Denglin Luo, Jinling Fan, Wenxue Zhu, Xinfang Liu, Qunyi Tong

**Affiliations:** 1 College of Food and Bioengineering, Henan University of Science and Technology, Henan Luoyang, China; 2 College of Chemistry and Chemical Engineering, Luoyang Normal University, Luoyang, China; 3 School of Food Science and Technology, Jiangnan University, Jiangsu Wuxi, China; Fujian Agriculture and Forestry University, CHINA

## Abstract

The effects of low molecular sugars (sucrose, glucose and trehalose) on the retrogradation of tapioca starch (TS) gels stored at 4°C for different periods were examined with different methods. Decrease in melting enthalpy (ΔHmelt) were obtained through differential scanning calorimetry analysis. Analysis of decrease in crystallization rate constant (k) and increase in semi-crystallization time (τ1/2) results obtained from retrogradation kinetics indicated that low molecular sugars could retard the retrogradation of TS gels and further revealed trehalose as the best inhibitor among the sugars used in this study. Fourier transform infrared (FTIR) analysis indicated that the intensity ratio of 1047 to 1022 cm^−1^ was increased with the addition of sugars in the order of trehalose > sucrose > glucose. Decrease in hardness parameters and increase in springiness parameters obtained from texture profile analysis (TPA) analysis also indicated that low molecular sugars could retard the retrogradation of TS gels. The results of FTIR and TPA showed a consistent sugar effect on starch retrogradation with those of DSC and retrogradation kinetics analysis.

## Introduction

Tapioca starch (TS) is one of the basic raw materials in food industry. It has been widely used as thickener, stabilizer or food matrix in food due to its physical properties of texture and stability, as well as its bland flavor [1]. However, during the process of transport or storage under refrigeration, the recrystallization of TS gel will influence the texture and mouth-feel of starchy food. In order to improve the taste of food, low molecular sugars are commonly used in starch-based food. At the same time, it was found that the addition of low molecular sugars could also influence gelatinization and retrogradation properties of starchy food [2]. Sugar addition has been reported to increase the tensile properties of membrane, and affect retrogradation and rheological properties of starch depending on the types and concentrations of both starch and sugar [3]. There are many kinds of starch and sugars used in food engineering, and large amount of experiments will be made to develop various starchy food formula, so it’s necessary to find more common or effective methods to evaluate the sugar effect on starch retrogradation.

Many kinds of methods have been used to evaluate starch retrogradation such as rheological method [4], X-ray diffraction method [5], thermal analysis method [6], and spectroscopic methods [7]. Differential scanning calorimetry (DSC) is a common used thermal analysis method which can effectively measure the degree of starch retrogradation. Fourier transform infrared (FTIR) spectroscopy can be used to evaluate the crystallization degree of starch within the scope of short range [8] because the intensity ratio of 1047/1022 cm^-1^ [R(1047/1022)] are sensitive to the change of starch structure. Texture profile analysis (TPA) is another important method because the hardness parameters are positively correlated and springiness parameters are negatively related to starch crystallization (retrogradation). DSC analysis is widely adopted to investigate the effect of low molecular sugars on starch retrogradation compared with rarely used FTIR and TPA methods [9, 10, 11].

In this paper, we studied the effects of different low molecular sugars (sucrose, glucose and trehalose) on TS retrogradation using DSC, FTIR and TPA methods under refrigeration. Considering the common transport or storage temperature for fresh products was 4°C, the experiments were operated at this temperature. And common sucrose, glucose and trehalose were used as additive to study the sugar effect on the retrogradation of TS gel. The FTIR and TPA methods will be evaluated when compared their results with that of DSC. This study will be conductive to our understanding of the interaction of starch and sugar during storage, as well as provide new evidence and effective methods for the development of starchy food.

## Materials and methods

### Materials

TS was supplied by Guangxi Mingyang Biochemical Technology Co., LTD, China. The moisture and amylose contents of TS were 13.41% and 17.10%, respectively. Analytical pure glucose, sucrose and trehalose were purchased from Sinopharm Group, China.

### Sample treatment

10% TS slurry was prepared in 250 ml beaker, and different contents (1 and 2 times of dry base starch) of glucose and trehalose were added, respectively. The mixture was stirred thoroughly and poured into a 20 mm-diameter plastic casing. Each casing was sealed at both ends, boiled in a saucepan for 30 minutes and then stored at 4°C fridge for 1, 3, 5, 7, 10, 14, 21 and 28 days, respectively.

### DSC analysis

The thermal properties were determined via DSC (PerkinElmer Pyris 1, Norwalk, CT). A small amount of sample (10–20mg) prepared in section 2.1 was taken out from the center of starchy sausage, put into DSC pan, weighed and sealed. An empty aluminum pan was used as a reference. Sample pans were heated at a rate of 10°C/min from 30 to 90°C. The melting initial temperature T_o_, peak temperature T_p_, end temperature T_e_ and melting enthalpy of each sample was calculated automatically from the curve. All results represent the average of records obtained from three independent experimental repeats.

The Avrami equation can be used to describe the kinetics of starch retrogradation. The model can be expressed as Eq 1:

$$\theta =(\Delta H_{\infty }-\Delta H_{t})/(\Delta H_{\infty }-\Delta H_{0})=\exp(-kt^{n})$$ (1)

Where *θ* is the fraction of uncrystallized starch at time *t*, Δ*H*_0_ and Δ*H*_t_ are the enthalpy changes at time 0 and time *t*, respectively, Δ*H*_∞_ is the limiting enthalpy change, *k* is the rate constant, and *n* is the Avrami exponent.

The values of *k* and *n* for the starch (where Δ*H*_0_ was zero in this study) were obtained from linear regression of the melting enthalpy data (Eq 2). τ_1/2_ is the half-time of starch retrogradation (Eq 3).

$$\ln[\text{-}\ln(\Delta H_{\infty })/(\Delta H_{\infty }\text{-}\Delta H_{t}]=n\ln t+\ln k$$ (2)

$$\tau_{1/2}={\left(\text{-}\ln0.5/k\right)}^{1/n}$$ (3)

### FTIR analysis

FTIR spectra were measured using an ABB BOMEN FTLA 2000–104 spectrometer. A small amount of sample was cut-off and washed four times with 80% methanol to completely wash-off the sugar. Each sample was prepared in KBr tablet and the FTIR spectrum was scanned during scan scope of 400–4000 cm^-1^. Each sample was scanned 32 times with a resolution of 2 cm^–1^. The spectra within 950–1200 cm^-1^ were linear baseline-corrected and deconvolution processed using the Omnic V.8 software. The half-bandwidth used for deconvolution was 26 cm^-1^, and the enhancement factor was 1.5. The infrared spectra were processed by a peak fitting procedure of origin 8.0 software. Peak positions and relative intensities, peak areas and full widths at half maximum (FWHM) of each peak were fit to compare the effects of low molecular sugars on starch retrogradation.

### TPA analysis

Texture property was measured using a TA-XT plus texture analyzer (TA-XTplus, Stable Microsystems, Surrey, UK). Each sample prepared in section 2.1 was sliced and cut off a 2cm-long cylinder, which was compressed axially in two consecutive cycles of 40% compression using an aluminium cylinder probe P/36 with pre-test speed of 1mm/s, test speed of 1.7 mm/s and post-test speed of 10 mm/s. From the force-deformation curves obtained, texture parameters of hardness and springness were computed using the Texture Expert software supplied with the instrument.

## The results and discussion

### Differential scanning calorimetry (DSC)

The effect of low molecular sugars on retrogradation properties of TS gel determined by DSC are summarized in Table 1. As shown in Table 1, *T*_*o*_, *T*_*p*_ and *T*_*c*_ of TS-sugar mixtures increased significantly with increasing sugar concentration at a given storage period, compared with that of the native starch. For example, pure TS gel displayed a peak temperature (*T*_*p*_) of 52.50°C after refrigeration of 7 days; the value of peak temperature (*T*_*p*_) increased to 63.41, 62.88 or 62.22°C when glucose, sucrose or trehalose was added into TS gel (Table 1). The increase of melting temperature by sugar effect coincided with the results of earlier researchers, which may result from the strong sugar-starch interactions, the reduction of available water content, change in the water structure or or “anti-plasticization” effects from the sugar-water co-solvent [12, 13].

**Table 1:** Retrogradation properties of TS gel without or with sugars after storage.

		*T*_*o*_ (°C)	*T*_*p*_ (°C)	*T*_*c*_ (°C)	*ΔHmelt* (J/g)
**3d**	**TS**	49.59 ± 0.02^g^	51.55 ± 0.03^k^	62.48 ± 0.03^k^	1.496 ± 0.02^j^
	**TS + glu**	60.47 ± 0.02^a^	67.35 ± 0.06^a^	74.91 ± 0.07^a^	1.350 ± 0.05^j^
	**TS +suc**	53.61 ± 0.12^c^	61.88 ± 0.15^ef^	67.86 ± 0.05^f^	1.337 ± 0.13^j^
	**TS + tre**	54.02 ± 0.03^b^	63.56 ± 0.03^b^	67.68 ± 0.22^f^	1.075 ± 0.05^k^
**7d**	**TS**	49.54 ± 0.05^g^	52.50 ± 0.03^i^	65.54 ± 0.05^h^	2.656 ± 0.02^g^
	**TS + glu**	50.54 ± 0.15^b^	63.41 ± 0.02^b^	69.39 ± 0.34^c^	2.352 ± 0.01^h^
	**TS +suc**	52.16 ± 0.03^d^	62.88 ± 0.04^c^	67.77 ± 0.06^f^	2.373 ± 0.02^h^
	**TS + tre**	48.50 ± 0.05^i^	62.22 ± 0.07^de^	65.56 ± 0.02^h^	2.015 ± 0.03^i^
**14d**	**TS**	44.00 ± 0.03^k^	51.57 ± 0.07^k^	63.13 ± 0.01^j^	4.686 ± 0.03^d^
	**TS + glu**	47.67 ± 0.14^j^	61.94 ± 0.40^def^	69.39 ± 0.021^c^	4.100 ± 0.06^e^
	**TS +suc**	49.61 ± 0.05^g^	59.20 ± 0.15^h^	67.57 ± 0.03^f^	4.186 ± 0.08^e^
	**TS + tre**	51.34 ± 0.36^e^	62.01 ± 0.04^de^	67.08 ± 0.02^g^	3.662 ± 0.13^f^
**21d**	**TS**	41.68 ± 0.03^l^	52.11 ± 0.04^j^	64.52 ± 0.05^i^	6.821 ± 0.08^a^
	**TS + glu**	51.03 ± 0.05^e^	62.31 ± 0.06^d^	74.00 ± 0.18^b^	5.850 ± 0.03^b^
	**TS +suc**	48.86 ± 0.05^h^	61.59 ± 0.11^fg^	68.97 ± 0.07^d^	5.999 ± 0.05^b^
	**TS + tre**	47.69 ± 0.09^j^	61.28 ± 0.06^g^	68.32 ± 0.02^e^	5.305 ± 0.05^c^

*T*_*o*_: onset temperature; *T*_*p*_, peak temperature; *T*_*c*_: conclusion temperature; *ΔHmelt*: Melting enthalpy; glu: glucose; suc: sucrose; tre: trehalose.

All tests were performed with three independent replicates. Mean ± standard deviation values in the same column followed by different letters are significantly different (*p* < 0.05) by Duncan’s test.

The melting enthalpy (ΔHmelt) of all samples increased significantly with increasing storage time. Under the same storage time, the melting enthalpy of TS gel decreased when different type of low molecular sugars was added. Furthermore, different low molecular sugars showed an obvious differences in decreasing the melting enthalpy of TS gel (trehalose > sucrose ≈ glucose). For example, adding trehalose could decrease the ΔHmelt of TS gel from 6.821 to 5.305 J/g when stored for 21 days, while sucrose or glucose could only decrease the ΔHmelt to 5.999 and 5.850 J/g, respectively. The difference in the decreased melting enthalpy demonstrates that trehalose is a fine candidate to delay or inhibit the recrystallization of TS gel.

### Retrogradation kinetics

The Avrami equation has been widely used to study the kinetics of starch retrogradation. The fitted kinetics parameters of TS gel without or with low molecular sugars are shown in Table 2. The determination coefficients (R^2^: 0.9822–0.9919) are over 0.98 demonstrating that the Avrami model agrees well with the thermal analysis data. The Avrami exponent (*n*) is viewed as a significant parameter which can reflect the mode of crystal nucleation. The Avrami exponent of TS gel without sugar or with low molecular sugars fall between 1.036–1.178, suggesting that the re-crystallization process follows the n = 1 type of one-dimensional rode-like growth from instantaneous nuclei [14, 15]. Statistically speaking, the addition of low molecular sugars doesn’t change the formation of crystal nucleus and the crystal growth mode of TS gel.

**Table 2:** Retrogradation kinetics parameters of TS gel without or with sugars.

	Avrami kinetic parameters			
	*n*	*k* (d^-1^)	*R*^*2*^	*τ*_*1/2*_ (d)
**TS**	1.067 ± 0.002^e^	0.036 ± 0.002^a^	0.9902	15.94 ± 0.12^g^
**TS + glu 2:1** **2:1**	1.043 ± 0.001^f^	0.036 ± 0.003^a^	0.9808	16.47 ± 0.16^f^
**TS + glu 1:1**	1.070 ± 0.003^d^	0.033 ± 0.002^b^	0.9919	17.26 ± 0.25^d^
**TS + suc 2:1** **2:1**	1.086 ± 0.002^b^	0.033 ± 0.005^b^	0.9840	16.65 ± 0.18^e^
**TS + suc 1:1**	1.072 ± 0.004^c^	0.032 ± 0.003^c^	0.9905	17.30 ± 0.26^c^
**TS + tre 2:1** **2:1**	1.036 ± 0.005^g^	0.036 ± 0.002^a^	0.9899	17.35 ± 0.33^b^
**TS + tre 1:1**	1.178 ± 0.002^a^	0.023 ± 0.001^d^	0.9822	18.20 ± 0.42^a^

*n*: Avrami exponent; *k*: crystallization rate constant; *τ*_*1/2*_: half crystallization-time.

The crystallization rate constant (*k*) and half-crystallization time (τ_1/2_) are also important parameters, reflecting the speed of crystallization [16, 17]. The *k* and τ_1/2_ value of TS gel is 0.036 d^-1^ and 15.94 d, respectively. When different low molecular sugars are added into TS gel, the *k* value is decreased and the τ_1/2_ value is increased obviously, illustrating the reducing recrystallization rate of TS gel during the process of cold storage. The more the amounts of low molecular sugars were added, the smaller the *k values were and* the higher the τ1/2 values were. That is to say, the recrystallization rate turned slower with the increase of sugar amounts. At the given sugar content, the τ1/2 values decrease according to the order of trehalose, sucrose and glucose, declaring adding low molecular sugars can inhibit the retrogradation property of TS gel in the same order. Meanwhile, the TS gel with low content of trehalose (TS: trehalose = 2: 1) had half-crystallization time longer than those of TS gel with higher content of sucrose or glucose (TS: sucrose = 1: 1, TS: glucose = 1: 1), indicating its better resistance to retrogradation. The sugar effect on starch retrogradation is agreement with that of our DSC results.

### Infrared spectra

**Fig 1:**
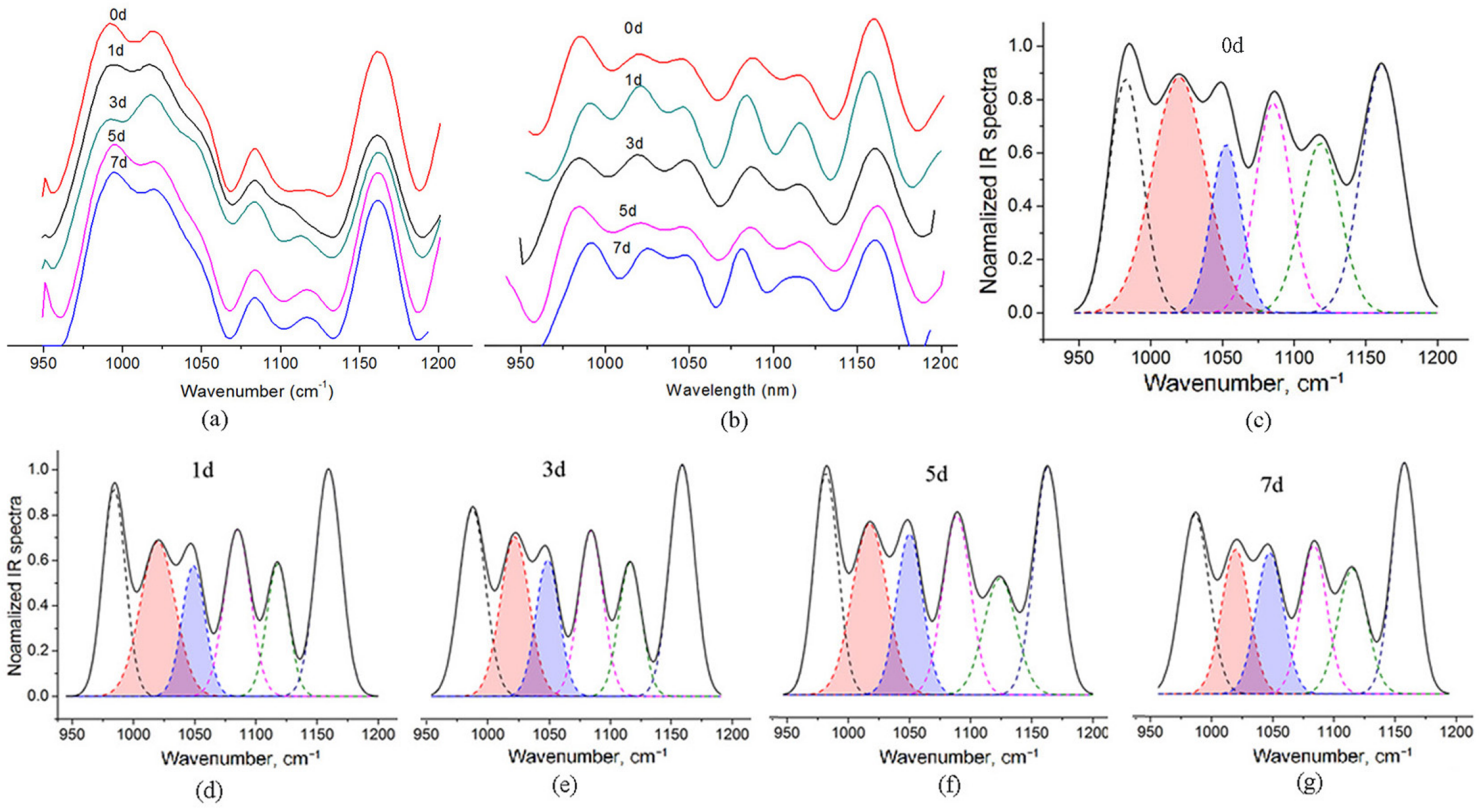
IR spectra of TS gel (10%) without sugars after refrigerated storage. (a) Original IR spectra (b) deconvoluted IR spectra (c) peak-fitted IR spectra.

The inhibitory effect of sugars on the retrogrdation of TS gel was evaluated by comparing the IR intensity ratio of 1047/1022 cm^−1^. The data process of TS gel without sugars was depicted detailed as a representative. The infrared spectra of TS gel (Fig 1A) display three main absorption bands in fingerprint region (950–1070, 1065–1135 and 1135–1185 cm^-1^). The intensity ratio of 1047/1022 cm^−1^ [*R*(1047/1022)] can express the short-range retrogradation of starch gel [18, 19] because the two absorbance peaks represent the amorphous and crystallization areas of starch, respectively. However, the overlap of related peaks makes the calculation of *R*(1047/1022) difficult. The overlapped peaks were separated by Fourier self-deconvolution (FSD) program of OMNIC software (Fig 1B) [20]. The integral intensity and width of each peak was fitted by multi-peak fitting program with Gaussian function of Origin 8.0 software [21] and the fitted spectra were shown in Fig 1C–1G. And thus, the *R*(1047/1022) and the short-range crystallization of TS gel can be clearly evaluated.

**Fig 2:**
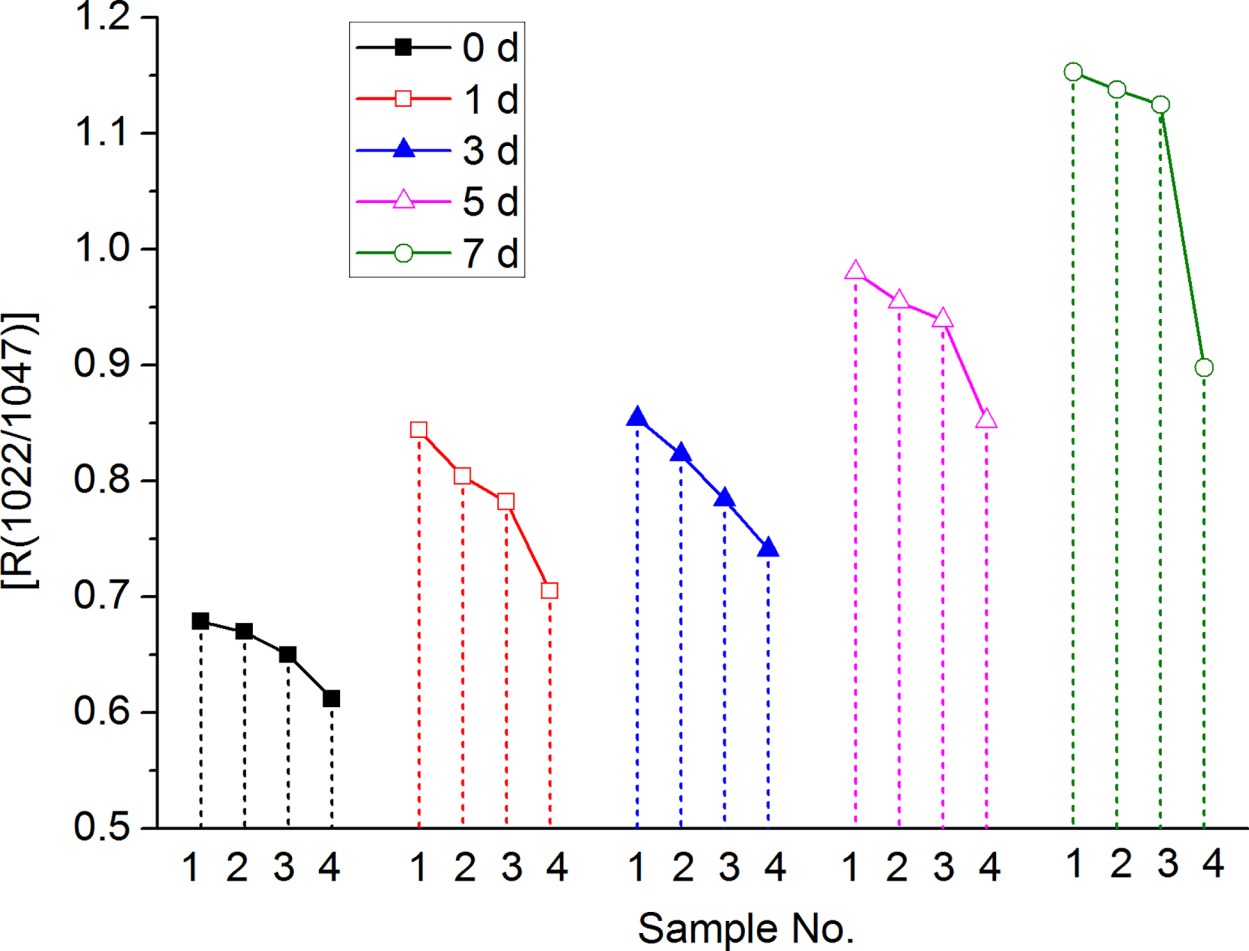
*R*(1047/1022) of TS gel (10%) without or with sugars after refrigerated storage. (Samples No. 1 is TS gel without sugars and sample Nos. 2, 3 and 4 are TS gel with glucose, sucrose and trehalose, respectively).

The *R*(1047/1022) of TS gel (10%) with low molecular sugars are also calculated with the same method and the results were shown in Fig 2. The figure showed that the *R*(1047/1022) of TS gel without or with low molecular sugars increased with the increase of cold storage time, indicating the gradual retrogradation of these samples. And the retrogradation effect was more apparent after storage 5 days. For the samples during the same storage period, the addition of low molecular sugars could decrease the *R*(1047/1022) of TS gel, indicating that sugars could prevent the retrogradation of TS gel. The inhibitory effect of low molecular sugars was consistent with the results of DSC analysis. For the TS gel with different sugars, the value of *R*(1047/1022) was decreased in the order of glucose, sucrose and trehalose. These results suggest that and trehalose is more effective to retard TS retrogradation than sucrose and glucose especially after storage for 7 days.

The effects of low molecular sugars is mainly due to the strong sugar-starch interactions between sugar and starch molecules chains, stabilizing the amorphous region of starch and inhibiting the crystallization of starch molecules in amorphous region. And during the process of cold storage, sugar molecules may be "involved in" the crystallization region of starch, and inhibit the crystallization of starch chains [22]. Compared with glucose and sucrose, trehalose can reduce the *R(1047/1022)* value of TS gel to a greater degree because trehalose has a larger number of procumbent hydroxy (trehalose 8.00, sucrose 6.30 and glucose 4.56) which is positively related to the starch-sugar interactions [23,24].

### Texture profile analysis (TPA)

**Fig 3:**
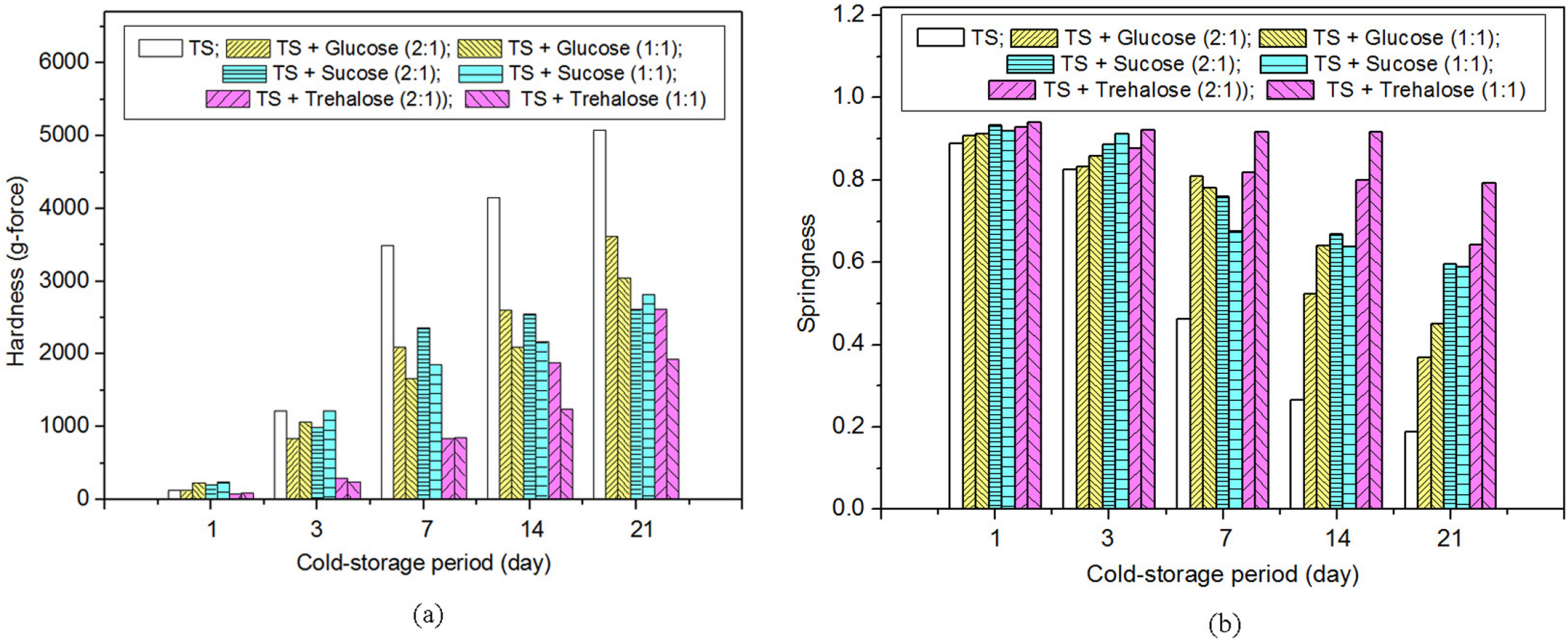
Hardness and springness of TS gels without or with sugars after storage.

Texture profile analysis (TPA) is another method to measure the property of starch. Hardness and springiness are two important TPA parameters. The texture changes of TS gel without or with different levels (2:1, 1:1, *w*:*w*) of low molecular sugars (trehalose, sucrose and glucose) are evaluated by TPA and the selected parameters (hardness and springiness) are shown in Fig 3.

Fig 3A shows that the hardness of TS gel without or with low molecular sugars increased with cold-storage period. Meanwhile, the addition of low molecular sugars decreased the hardness obviously, especially for the samples with long-term storage. However, more sugar could decrease the hardness of TS gel to a small degree, even not change or increase the hardness for a relatively short storage period. Specifically, when TS gel with glucose or sucrose was stored for 1 or 3 days, the hardness of high sugar TS gel is higher than that of low level sugar TS gel; when above-mentioned samples were stored more than 7 days, the results were opposite. The increase of hardness of high-sugar TS gel might be caused by the decrease of “available water” due to moisture binding by sugars, inhibition of granular hydration and sucrose-starch interactions [25]. During the same storage period, trehalose could decrease the hardness of TS gels to a greater extent compared with sucrose and glucose, indicating the high anti-retrogradation activity of trehalose.

Springness is another TPA parameter which represents the activity of TS gel returning to normal when the deformation force is removed. Fig 3B shows that the springiness of TS gels without sugar decrease obviously (from 0.89 to 0.19) with the extension of cold storage time (stored from 0 to 21 days). The decreasing springiness observed in TS gel could be affected by adding low molecular sugars. During the same storage period, the springiness of TS gel with low molecular sugars is larger than that of TS gel, and the sugar effect is more obvious after storage more than 7 days. Compared with sucrose and glucose, trehalose is a bit easier to keep the stability of springiness. For example, the springiness of TS gel with high content of trehalose (TS:Tre = 1:1, w:w) has decreased just 2.4% and 15.7% after storage of 14 days and 21 days, respectively; the springiness of TS gel with the same content of sucrose or glucose decreased 30.5% or 29.9% after storage of 14 days, and declined 36.0% or 50.6% after storage of 21 days. Therefore, trehalose can inhibit the decrease of springiness of TS gel to a greater extent.

## Conclusion

According to the results of DSC, retrogradation kinetics analysis, FTIR and TPA, low molecular sugars could inhibit the retrogradation of TS gels. Among the three sugars, trehalose had a greater effect on melting enthalpy (ΔHmelt), crystallization rate constant (*k*), semi-crystallization time (*τ*_1/2_), *R*(1047/1022), hardness and springiness parameter than sucrose and glucose. Therefore, trehalose had greater anti-retrogradation effect on TS gels than sucrose or glucose. The consistent results of different methods prove the availability of FTIR and TPA to study the sugar effect on starch retrogradation compared with the common used DSC and retrogradation kinetics analysis.
